# Hemifield-based analysis of pattern electroretinography in normal subjects and patients with preperimetric glaucoma

**DOI:** 10.1038/s41598-024-55601-9

**Published:** 2024-03-01

**Authors:** Eun Jung Ahn, Young In Shin, Young Kook Kim, Jin Wook Jeoung, Ki Ho Park

**Affiliations:** 1 Department of Ophthalmology, Seoul National University College of Medicine, Seoul, Korea; 2 Daehakro Seoul Eye Clinic, Seoul, Korea; 3 Department of Ophthalmology, Gachon University Gil Hospital, Incheon, Korea; 4 Department of Ophthalmology, Seoul National University Hospital, Seoul, Korea

**Keywords:** Visual system, Pattern vision, Eye diseases

## Abstract

This prospective cross-sectional study investigated the visual function of preperimetric glaucoma (PPG) patients based on hemifield (HF) pattern electroretinogram (PERG) amplitudes. Thirty-two (32) normal subjects and 33 PPG patients were enrolled in control and PPG groups, respectively. All of the participants had undergone full ophthalmic examinations, including spectral-domain optical coherence tomography (SD-OCT), visual field (VF) examination and pattern electroretinography (PERG). The PERG parameters along with the HF ratios of SD-OCT and PERG were compared between the control and PPG groups. Pairwise Pearson's correlation coefficients and linear regression models were fitted to investigate the correlations. The PERG N95 amplitudes were significantly lower in the PPG group (*P* < 0.001). The smaller/larger HF N95 amplitude ratio of the PPG group was found to be smaller than that of the control group (0.73 ± 0.20 vs. 0.86 ± 0.12; *P* = 0.003) and showed positive correlations with affected HF average ganglion cell-inner plexiform layer (GCIPL) thickness (r = 0.377, *P* = 0.034) and with average GCIPL thickness (r = 0.341, *P* = 0.005). The smaller/larger HF N95 amplitude ratio did not significantly change with age (β =  − 0.005, *P* = 0.195), whereas the full-field N95 amplitude showed a negative correlation with age (β =  − 0.081, *P* < 0.001). HF analysis of PERG N95 amplitudes might be particularly useful for patients with early glaucoma.

## Introduction

Glaucoma is a group of optic neuropathies characterized by malfunction and death of retinal ganglion cells (RGCs)^1^. The diagnostic strategy for glaucoma is focused on identification of structural and functional changes related to RGC damage. Perimetry is used for a functional test, while fundus photography and/or optical coherence tomography (OCT) is applied to assess the optic nerve head (ONH) and peripapillary retinal nerve fiber layer (RNFL)^2,3^.

Pattern electroretinography (PERG) is a glaucoma test that measures the function of the inner-retinal structures^4,5^. It is relatively objective and can detect dysfunction before structural abnormalities are visible^6–8^. In contrast, standard automated perimetry (SAP) is a subjective test and includes the integrated activity of neural structures beyond the retina. Because glaucomatous visual field (VF) defects do not appear on SAP until 25–35% of RGCs have been lost ^6,8,9^, preperimetric glaucoma (PPG) patients often have neuroretinal rim loss in the ONH or focal RNFL defect but with a normal VF by SAP ^10^. A longitudinal study found that PERG changes show defects in glaucoma suspects 4 years before they are apparent on SAP^11^.

Going beyond the previous studies, the present investigation focused on hemifield (HF) PERG amplitude and its ratio to determine if it could be used to detect early functional defects in glaucoma patients. Accordingly, we compared PERG parameters in PPG patients and normal subjects and evaluated the diagnostic utility of HF-based analysis of PERG parameters. By honing in on these parameters, we aim to contribute to the refinement of diagnostic strategies for detecting glaucoma at its incipient stages.

## Results

The data were evaluated for 33 eyes of 33 PPG patients who had met the inclusion criteria and 32 eyes of 32 normal subjects. Table 1 compares the demographics and clinical characteristics of the PPG patients with those of the normal controls. There was no statistically significant difference in the two groups’ age, gender, best-corrected visual acuity (BCVA), spherical equivalent, central corneal thickness, or axial length (AXL), except for intraocular pressure (IOP). The IOP value of the PPG group was lower than that of the normal controls, and this might have been due to the fact that 12 PPG patients (36.4%) were taking topical glaucoma medications. The PPG group showed significantly thinner RNFL and ganglion cell-inner plexiform layer (GCIPL) thickness compared with the normal controls (both *P* < 0.001), and there were no significant intergroup differences in the SAP parameters.

**Table 1:** Participants’ characteristics.

Characteristics	Control group (N = 32)	Preperimetric glaucoma group (N = 33)	*P*-value
Age (years)	48.63 ± 7.30	52.58 ± 10.57	0.085^a^
Gender (n, %)			
Male	13 (40.6)	19 (57.6)	0.172^b^
Female	19 (59.4)	14 (42.4)	
BCVA (logMAR)	0.00 ± 0.02	− 0.03 ± 0.10	0.419^c^
Spherical equivalent	− 2.54 ± 2.67	− 3.31 ± 2.44	0.254^a^
Central corneal thickness (μm)	547.50 ± 29.45	536.48 ± 29.97	0.161^a^
Axial length (mm)	24.36 ± 1.63	25.13 ± 1.60	0.105^a^
Intraocular pressure (mmHg)	15.53 ± 2.79	13.79 ± 3.06	**0.020**^**a**^
SD-OCT			
Average RNFL thickness (μm)	94.69 ± 10.07	77.79 ± 8.77	** < 0.001**^a^
Average GCIPL thickness (μm)	81.53 ± 5.39	73.39 ± 6.13	** < 0.001**^a^
SAP			
MD (dB)	− 1.73 ± 1.30	− 1.19 ± 1.30	0.095^a^
PSD (dB)	1.66 ± 0.37	1.83 ± 0.50	0.131^a^
Affected HF average total deviation (dB)	N/A	− 1.24 ± 1.67	N/A

Values are mean ± standard deviations.

Bold indicates that the P value reached statistical significance (< 0.05).

BCVA, best-corrected visual acuity; logMAR, logarithm of the minimum angle of resolution; SD-OCT, spectral-domain optical coherence tomography; RNFL, retinal nerve fiber layer; GCIPL, ganglion cell–inner plexiform layer; SAP, standard automated perimetry; MD, mean deviation; PSD, pattern standard deviation; HF, hemifield.

^a^Student *t* test.

^b^Chi-square test.

^c^Mann–Whitney test.

A comparison of the PERG parameters between the control and PPG groups is provided in Table 2. The full field (FF), upper hemifield (UF), and lower hemifield (LF) N95 amplitudes were significantly lower in the PPG group (*P* < 0.001, *P* < 0.001, and *P* = 0.009), and the FF P50 amplitude was also significantly lower in the PPG group (*P* = 0.013). The HF ratios of the spectral-domain optical coherence tomography (SD-OCT) and PERG parameters are listed in Table 3. The PPG group showed significantly lower thinner/thicker HF RNFL and GCIPL thickness ratios compared with the control group (0.85 ± 0.11 vs. 0.92 ± 0.06, 0.91 ± 0.09 vs. 0.98 ± 0.02; *P* = 0.002 and *P* < 0.001, respectively). The smaller/larger HF N95 amplitude ratio of PERG was significantly lower among the PPG group (0.74 ± 0.21) than among the control group (0.86 ± 0.12; *P* = 0.007). For the PPG patients, the affected/unaffected HF N95 amplitude ratio was 0.89 ± 0.39.

**Table 2:** Pattern electroretinogram (PERG) parameters in participants.

Characteristics	Control group (N = 32)	Preperimetric glaucoma group (N = 33)	*P*-value
PERG N95 Amplitude (μV)			
Full field (FF)	6.62 ± 1.26	5.24 ± 1.62	** < 0.001**
Upper field (UF)	3.30 ± 0.74	2.52 ± 1.01	**0.001**
Lower field (LF)	3.53 ± 0.76	2.93 ± 1.02	**0.009**
PERG P50 Amplitude (μV)			
Full field (FF)	3.65 ± 0.98	2.93 ± 1.25	**0.013**
Upper field (UF)	1.87 ± 0.57	1.62 ± 0.76	0.136
Lower field (LF)	2.00 ± 0.57	1.88 ± 0.81	0.484
PERG N95 Amplitude ratio			
UF/FF	0.51 ± 0.11	0.49 ± 0.14	0.602
LF/FF	0.54 ± 0.11	0.57 ± 0.14	0.792
UF/LF	0.95 ± 0.21	0.93 ± 0.46	0.913
LF/UF	1.10 ± 0.23	1.28 ± 0.65	0.143
PERG P50 Amplitude ratio			
UF/FF	0.55 ± 0.23	0.61 ± 0.32	0.404
LF/FF	0.60 ± 0.29	0.70 ± 0.34	0.209
UF/LF	0.99 ± 0.37	0.94 ± 0.43	0.583
LF/UF	1.18 ± 0.64	1.45 ± 1.24	0.276

Values are mean ± standard deviations.

Bold indicates that the P value reached statistical significance (< 0.05).

PERG, pattern electroretinogram; FF, full field; UF, upper field; LF, lower field.

**Table 3:** Comparison of Hemifield (HF) ratios of spectral-domain optical coherence tomography (SD-OCT) and pattern electroretinogram (PERG) parameters in normal controls and patients with preperimetric glaucoma (PPG).

Characteristics	Control group (N = 32)	Preperimetric glaucoma group (N = 33)	*P*-value
SD-OCT			
Affected/Unaffected HF RNFL thickness ratio	N/A	0.86 ± 0.12	N/A
Affected/Unaffected HF GCIPL thickness ratio	N/A	0.92 ± 0.09	N/A
Thinner/Thicker HF RNFL thickness ratio	0.92 ± 0.06	0.85 ± 0.11	**0.002**
Thinner/Thicker HF GCIPL thickness ratio	0.98 ± 0.02	0.91 ± 0.09	** < 0.001**
PERG			
Affected/Unaffected HF N95 amplitude ratio	N/A	0.89 ± 0.39	N/A
Affected/Unaffected HF P50 amplitude ratio	N/A	1.07 ± 0.60	N/A
Smaller/Larger HF N95 amplitude ratio	0.86 ± 0.12	0.74 ± 0.21	**0.007**
Smaller/Larger HF P50 amplitude ratio	0.76 ± 0.16	0.74 ± 0.26	0.697

Affected HF was defined as a localized one-HF with localized RNFL defect or glaucomatous optic disc changes.

Values are mean ± standard deviations.

Bold indicates that the P value reached statistical significance (< 0.05).

HF, hemifield; SD-OCT, spectral-domain optical coherence tomography; PERG, pattern electroretinogram; PPG, preperimetric glaucoma; RNFL, retinal nerve fiber layer; GCIPL, ganglion cell–inner plexiform layer.

The outcome of the pairwise correlation analysis between the PERG and SD-OCT parameters is shown in Table 4. The FF N95 amplitude showed a positive correlation with the average RNFL thickness (r = 0.351, *P* = 0.004). There was a borderline significant positive correlation (r = 0.242, *P* = 0.052, post-hoc power = 0.499) between the average RNFL thickness and the smaller/larger HF N95 amplitude ratio. The FF, affected HF, and smaller/larger HF N95 amplitudes exhibited positive correlations with both the average and affected HF average GCIPL thicknesses (Table 4). There was no significant association between the SAP parameters and any of the PERG parameters. Supplementary Fig. 1 shows the scatterplots and results of those pairwise correlation analyses.

**Table 4:** Correlation between pattern electroretinogram (PERG) parameters and spectral-domain optical coherence tomography (SD-OCT)/standard automated perimetry (SAP) parameters.

	FF PERG N95 amplitude		Affected HF PERG N95 amplitude		Smaller/larger HF N95 amplitude ratio	
	r	*P*-value	r	*P*-value	r	*P*-value
Average RNFL thickness	0.351	**0.004**	0.237	0.184	0.242	0.052
Affected HF average RNFL thickness	0.164	0.361	0.253	0.155	0.047	0.793
Average GCIPL thickness	0.382	**0.002**	0.435	**0.011**	0.341	**0.005**
Affected HF average GCIPL thickness	0.442	**0.010**	0.507	**0.003**	0.377	**0.034**
SAP MD	− 0.143	0.256	− 0.223	0.213	− 0.060	0.637
Affected HF average total deviation	− 0.097	0.596	− 0.233	0.200	0.008	0.966

Values are mean ± standard deviations.

Bold indicates that the P value reached statistical significance (< 0.05).

PERG, pattern electroretinogram; SD-OCT, spectral-domain optical coherence tomography; SAP, standard automated perimetry; FF, full field; HF, hemifield; RNFL, retinal nerve fiber layer; GCIPL, ganglion cell–inner plexiform layer; MD, mean deviation.

Table 5 displays the area under the receiver operating characteristic curves (AUROC) in terms of quantitative parameters. Among the various parameters, the inferior average RNFL thickness (0.910) showed the best diagnostic performance. The AUROC of each SD-OCT/SAP and PERG P50 parameter was compared with those of the PERG N95 parameters for the purpose of trying to distinguish between normal and PPG eyes (Table 5). The AUROCs of average RNFL thickness were significantly greater than those of PERG N95 amplitude in the total, UF, and LF analyses (*P* = 0.026, *P* = 0.019, and *P* = 0.031, respectively). However, the thinner/thicker HF RNFL thickness ratio was not significantly greater than the smaller/larger HF N95 amplitude ratio (*P* = 0.822), and the thinner/thicker HF GCIPL thickness ratio was significantly greater than the smaller/larger HF N95 amplitude ratio (*P* = 0.031). The AUROC of PERG N95 amplitude was greater than that of PERG P50 amplitude for the LF analysis (*P* = 0.033). The AUROC curves for differentiating PPG eyes from normal control eyes are displayed in Supplementary Fig. 2.

**Table 5:** Areas under the receiver operating characteristic curve (AUROCs) for discrimination of preperimetric glaucoma (PPG) from normal control.

	AUROC	95% CI		*P*-value
		Lower bound	Upper bound	
SD-OCT average RNFL thickness				
Total	0.898	0.824	0.972	**0.026**^a^
Superior	0.846	0.751	0.941	**0.031**^b^
Inferior	0.910	0.841	0.979	**0.019**^c^
SD-OCT thinner/thicker HF RNFL thickness ratio	0.687	0.552	0.821	0.822^d^
SD-OCT average GCIPL thickness				
Total	0.838	0.742	0.933	0.186^a^
Superior	0.797	0.688	0.906	0.190^b^
Inferior	0.806	0.704	0.909	0.396^c^
SD-OCT thinner/thicker HF GCIPL thickness ratio	0.834	0.737	0.931	**0.031**^d^
SAP MD	0.384	0.247	0.521	0.188^a^
SAP PSD	0.589	0.448	0.729	0.104^a^
PERG N95 Amplitude				
Full field	0.738	0.615	0.861	N/A
Upper field	0.744	0.622	0.866	N/A
Lower field	0.689	0.560	0.819	N/A
PERG smaller/larger HF N95 amplitude ratio	0.663	0.530	0.796	N/A
PERG P50 Amplitude				
Full field	0.680	0.546	0.814	0.103^a^
Upper field	0.619	0.481	0.756	0.202^c^
Lower field	0.582	0.439	0.726	**0.033**^b^
PERG smaller/larger HF P50 amplitude ratio	0.534	0.391	0.677	0.175^d^

Bold indicates that the P value reached statistical significance (< 0.05).

AUROC, Area Under the Receiver Operating Characteristic Curve; PPG, preperimetric glaucoma; CI, confidence interval; SD-OCT, spectral-domain optical coherence tomography; RNFL, retinal nerve fiber layer; HF, hemifield; GCIPL, ganglion cell–inner plexiform layer; SAP = standard automated perimetry; MD, mean deviation; PSD, pattern standard deviation; PERG, pattern electroretinogram.

^a^P value: Comparison of each parameter with FF PERG N95 amplitude.

^b^P value: Comparison of each parameter with LF PERG N95 amplitude.

^c^P value: Comparison of each parameter with UF PERG N95 amplitude.

^d^P value: Comparison of each parameter with smaller/larger HF PERG N95 amplitude ratio.

**Figure 1:**
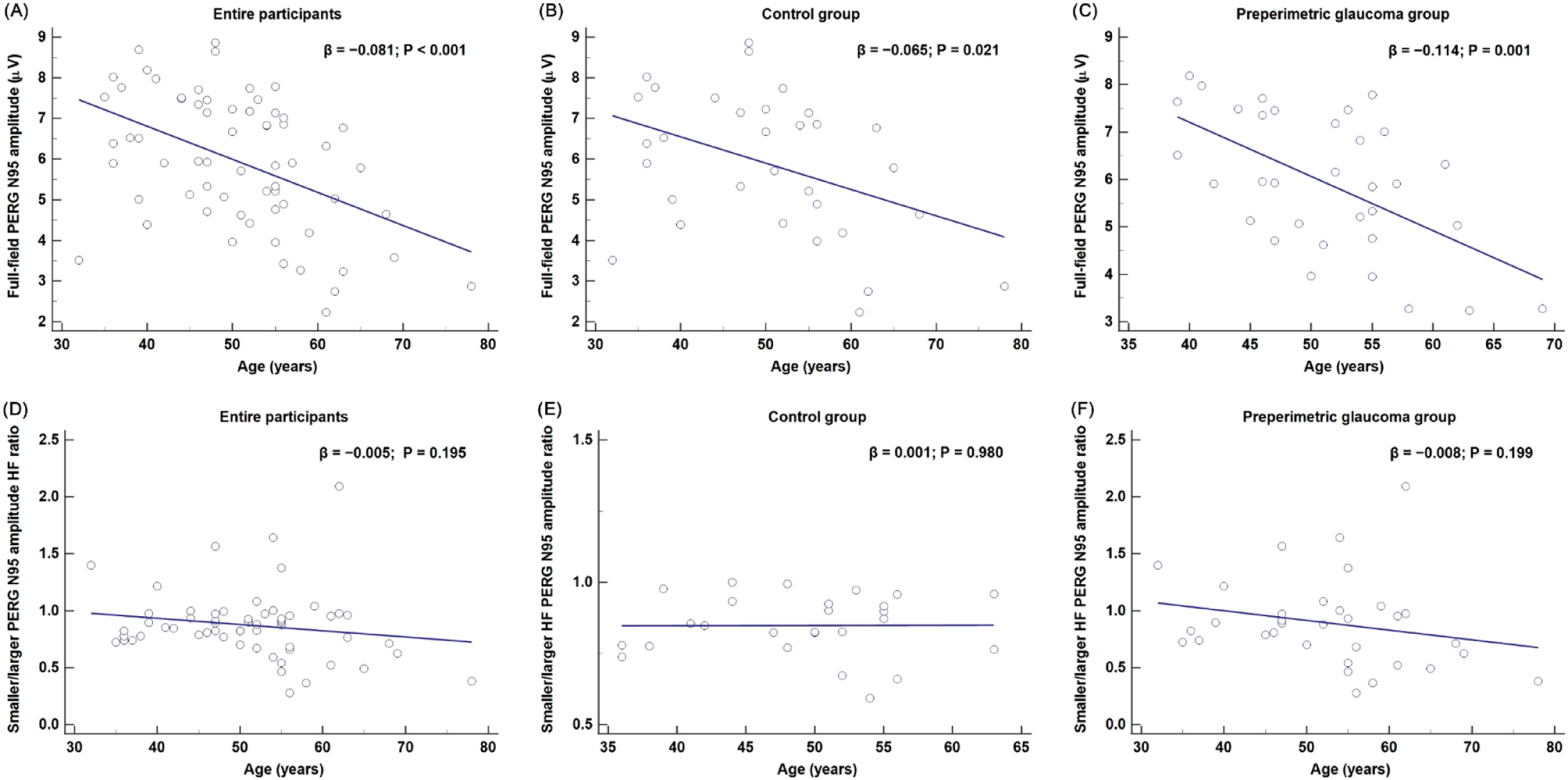
Scatterplot and linear regression model showing correlation between age and pattern electroretinogram (PERG) N95 amplitude. Full-field (FF) PERG N95 amplitude was negatively correlated with age, with a regression coefficient of − 0.460 (β =  − 0.081, P < 0.001, (**A**)) in the entire study participants and also negatively correlated with age in the control and preperimetric glaucoma (PPG) groups (β =  − 0.065, P = 0.021, (**B**); β =  − 0.114, P = 0.001, (**C**), respectively). In contrast, the smaller/larger hemifield (HF) N95 amplitude ratio showed no correlation with age for the entire participants (β =  − 0.005, P = 0.195, (**D**)), the control group (β = 0.001, P = 0.980, (**E**)) or the PPG group (β =  − 0.008, P = 0.199, (**F**)).

**Figure 2:**
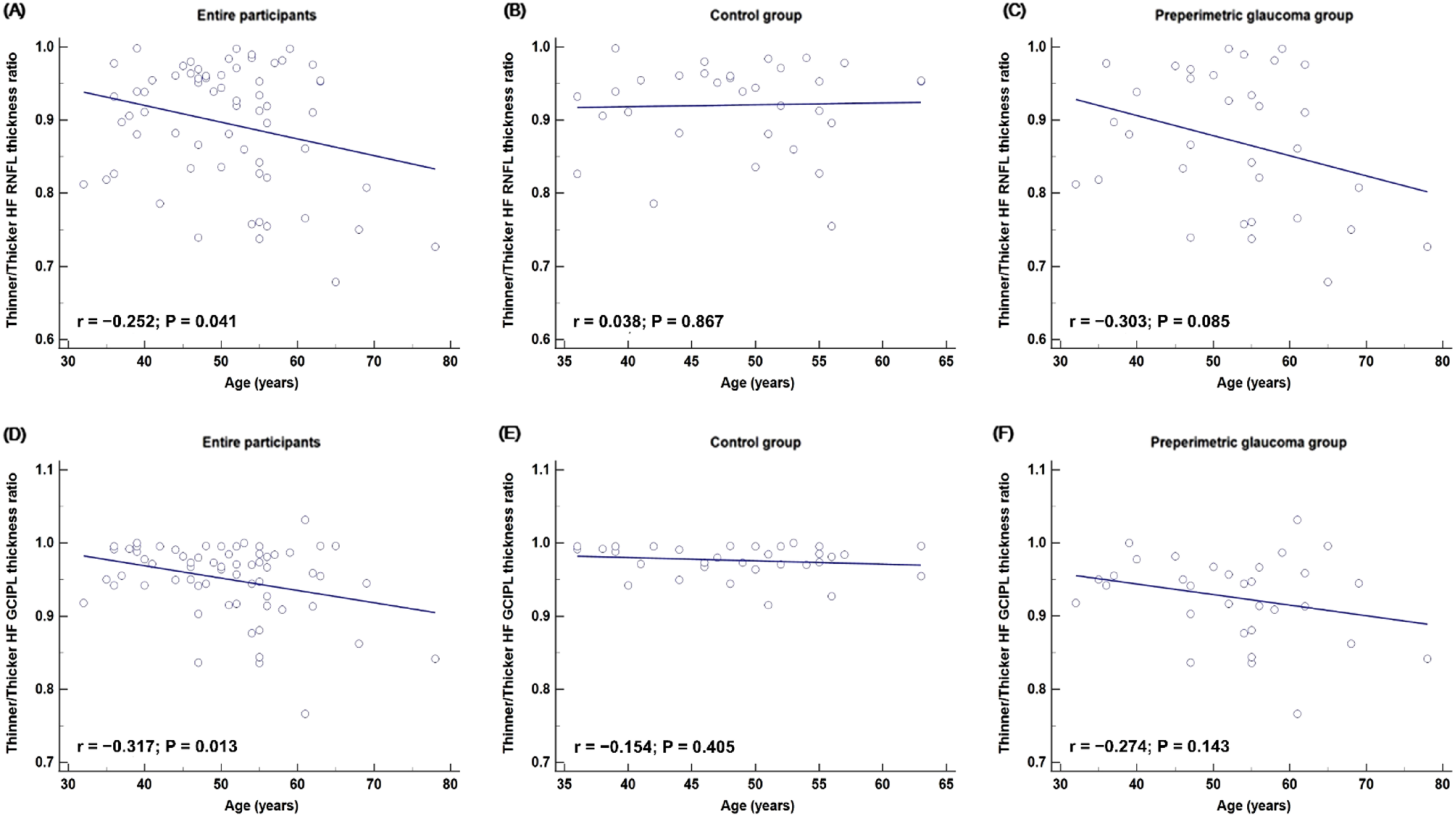
Scatterplots showing correlation between age and spectral-domain optical coherence tomography (SD-OCT) hemifield parameters. Entire study subjects showed a negative correlation between age and the thinner/thicker hemifield (HF) retinal nerve fiber layer (RNFL) and ganglion cell–inner plexiform layer (GCIPL) thickness ratios (r =  − 0.252, P = 0.041, (**A**); r =  − 0.317, P = 0.013, (**D**)). However, there was no age correlation seen in the thinner/thicker HF thickness ratio for RNFL and GCIPL parameters in the subgroup analyses for the PPG and control groups (all P > 0.05, (**B**, **C**, **E**, **F**)).

Scatter plots of FF PERG N95 amplitude against age for the entire study participants and control and PPG groups revealed amplitude decreases (Fig. 1A–C). Correspondingly, the linear regression models showed a significant negative correlation of age with FF PERG N95 amplitude (β =  − 0.081, 95% CI − 0.12 to − 0.04, *P* < 0.001, Fig. 1A) in the entire population. The control and PPG groups also exhibited negative correlations between age and the FF PERG N95 amplitude (β =  − 0.065, *P* = 0.021, Fig. 1B; β =  − 0.114, *P* = 0.001, Fig. 1C). However, age had no discernible impact on the smaller/larger HF N95 amplitude ratio in the entire population (β =  − 0.005, *P* = 0.195, Fig. 1D) or in either the control or the PPG group (β = 0.001, *P* = 0.980, Fig. 1E; β =  − 0.008, *P* = 0.199, Fig. 1F). We additionally investigated scatterplots of the relationship between age and the HF parameters of SD-OCT. Age and thinner/thicker HF RNFL and GCIPL thickness ratios were correlated across the board in the entire study participants (r =  − 0.252, *P* = 0.041, Fig. 2A; r =  − 0.317, *P* = 0.013, Fig. 2D). By contrast, no age correlation was observed in the RNFL and GCIPL measures' thinner/thicker HF thickness ratio in the subgroup analyses for the PPG and control groups (all *P* > 0.05, (Fig. 2B, C, E, F).

**Figure 3:**
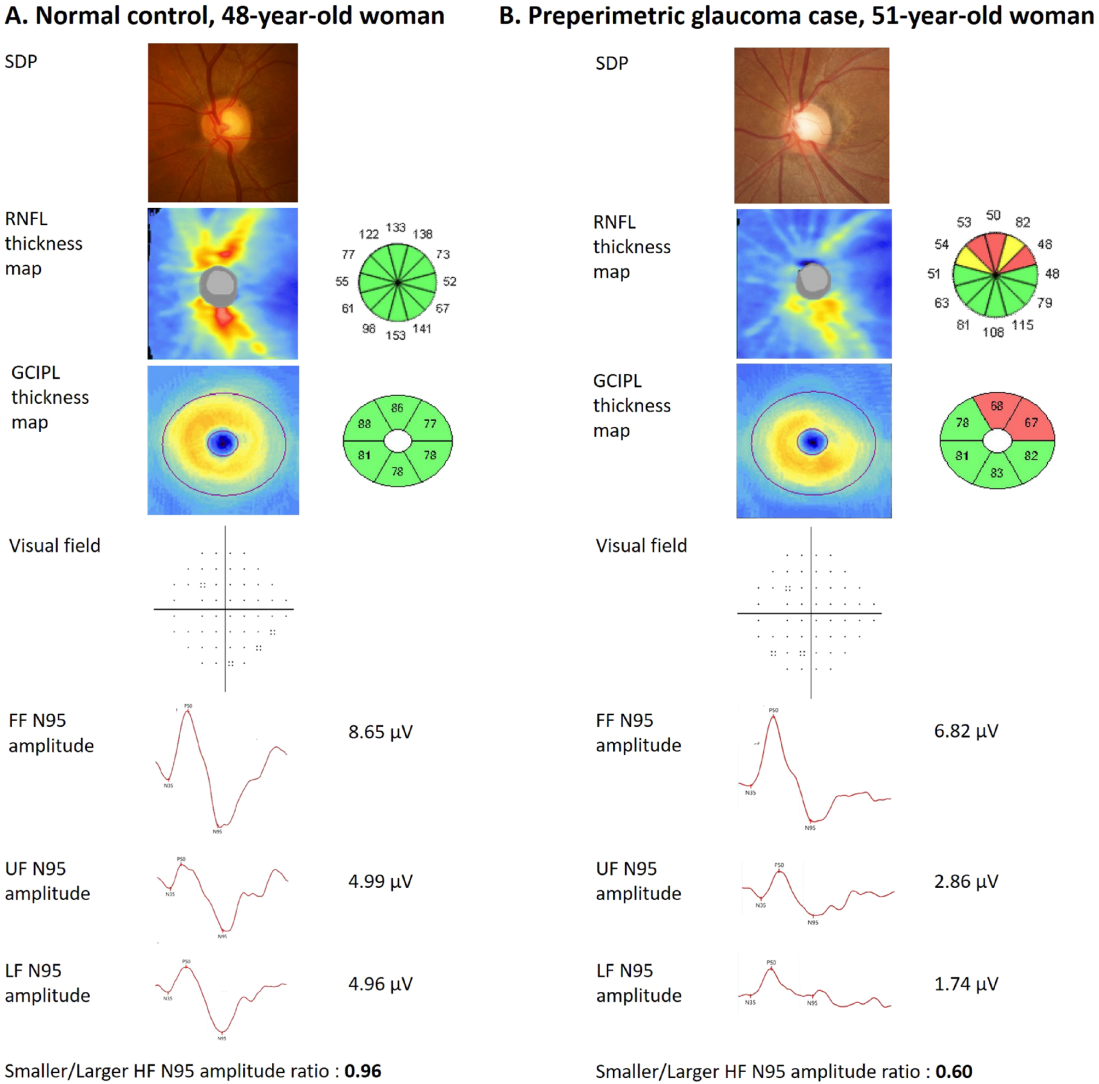
Representative cases. (**A**) Normal control case of 48-year-old woman. (**B**) Preperimetric glaucoma (PPG) case of 51-year-old woman. (**A**) Stereoscopic disc photography (SDP) revealed no glaucomatous optic disc rim change in the normal eye of a 48-year-old female control, and optical coherence tomography (OCT) thickness maps revealed no ganglion cell inner plexiform layer (GCIPL) or retinal nerve fiber layer (RNFL) thinning with normal visual field. (**B**) SDP revealed optic disc rim loss in the superotemporal area, and OCT exhibited thinning in the superior region of the RNFL and GCIPL thickness maps with normal visual field test findings. The PPG case showed decreased PERG N95 amplitudes in the full field (FF), upper field (UF), and lower field (LF). The smaller/larger HF N95 amplitude ratio was 0.60 in the PPG case and was lower than the value for the normal control (0.96).

Representative cases of normal control and PPG eyes are presented in Fig. 3. A normal control case of a 48-year-old woman (A) showed no glaucomatous neuroretinal rim change on stereoscopic disc photography (SDP), and SD-OCT RNFL and GCIPL thickness maps revealed no thinning. Also, no glaucomatous defect was found on the Humphrey VF test. In a 51-year-old woman with PPG (B), SDP revealed superior rim narrowing, and SD-OCT RNFL/GCIPL thickness analyses showed superior defect. On the Humphrey VF test, no glaucomatous defect was discovered. However, the FF, UF, and LF PERG N95 amplitudes were lower than in the normal control case. The smaller/larger HF N95 amplitude ratio in the PPG case was 0.60 and was lower than that for the normal control (0.96).

## Discussion

In the PPG group, there were statistically significant decreases in the FF, UF, and LF PERG N95 amplitudes and smaller/larger HF N95 amplitude ratio relative to the normal control group (FF, UF: both *P* < 0.001, LF: *P* = 0.009, smaller/larger HF N95 amplitude ratio: *P* = 0.007). The average GCIPL thickness was positively correlated with the FF PERG N95 amplitude, affected HF N95 amplitude, and smaller/larger HF N95 amplitude ratio (r = 0.382, *P* = 0.002; r = 0.435,* P* = 0.011; r = 0.341, *P* = 0.005), as was the affected HF average GCIPL thickness (r = 0.442, *P* = 0.010; r = 0.507, *P* = 0.003; r = 0.377, *P* = 0.034).

It is understood that PERG mostly reflects RGC functions, with some contribution from other retinal cells ^12^. Accordingly, in terms of distinguishing glaucoma patients from healthy controls, PERG demonstrated positive results ^13–16^. Previous studies have found that PERG amplitudes were attenuated even in glaucoma suspect or PPG patients ^15,17^. Among other PERG parameters, such as P50 amplitude or implicit time, N95 amplitude is the most sensitive for glaucoma evaluation ^18–21^; and in fact, Jung et al.^10^ recently emphasized its usefulness as an ancillary tool for assessment of visual function in PPG patients with RNFL loss. Transient PERG records the responses of both the ON and OFF pathways of RGCs ^22^, with the N95 amplitude reflecting spiking activity and the P50 amplitude reflecting non-spiking activity ^23^. On the other hand, steady-state PERG records only ON pathway activity ^22^. The P50 component is believed to have contributions from the outer and middle retina, whereas N95 and steady-state PERG are associated more with the RGCs ^24,25^. Owing to these differences, the N95 amplitude should be more sensitive for glaucoma patients, and thus, we investigated its applicability to PPG patients in this study.

We hypothesized that utilizing the HF PERG N95 amplitudes and their ratios might result in more clinically useful measurements for the visual function of PPG patients than could be obtained from the FF PERG N95 amplitude alone. Our reasoning was based on the consideration that the additional data on RGC activity asymmetry revealed by comparing the upper and lower fields would prove constructive to the understanding of glaucomatous asymmetry involving the horizontal meridian. Graham et al.^26^ demonstrated that the upper-to-lower HF PERG ratio is slightly less than 1.0 and remains stable with age in normal subjects. Salgarello et al.^25^ noted that this ratio had good diagnostic accuracy when comparing normal controls with localized perimetric glaucoma patients. In the present study, we assumed that there would be electrophysiologic or functional differences in the location of the dominant HF as well as in the location of the affected HF for each subject in both the normal and PPG groups. In order to compare the normal controls with the PPG group, we determined the HF ratio of smaller/larger PERG N95 amplitude in each patient. This was done due to the impossibility of calculating the affected/unaffected HF ratio for the normal controls. The smaller/larger HF N95 amplitude ratio was significantly lower for the PPG group, and moreover, was in accordance with the other, SD-OCT-based parameters (Table 3). A plot of subject age and FF PERG N95 amplitude revealed that amplitude decreased with age (Fig. 1A–C). However, age had no discernible impact on either group's smaller/larger HF PERG N95 amplitude (Fig. 1D–F). In contrast to the PERG results, it was confirmed that age had an impact on the HF ratio in terms of the SD-OCT RNFL/GCIPL parameters (Fig. 2). Both the upper and lower HFs may be equally affected by age-related PERG amplitude decline, and the HF ratio may stay constant ^26,27^. In fact, the ratio could resolve many confounding issues that come up when evaluating PERG results in glaucoma patients. The effect of age on PERG N95 amplitude can be eliminated by using the ratio of the HF amplitude. Thus, this HF ratio may be useful as a biomarker for early identification of functional abnormalities in early glaucoma ^28^. But there is a chance that increasing damage in both the upper and lower HF will eventually have an impact on the HF ratio, which could lessen its importance. Given this, we suggest that a different approach, one that applied the z-score from the baseline to the PERG follow-up evaluations in a recent study, be utilized ^29^. This method may be useful in measuring the topographic distribution of damage as well as the disease course. PERG certainly has the potential to serve as a valuable biomarker for glaucoma progression as well as for long-term monitoring of glaucoma in large-scale prospective studies.

Gordon et al.^30^ found that PERG amplitude and RNFL thickness were both significantly correlated with SAP mean deviation (MD) in early-treated glaucoma patients. Jung et al.^11^ reported that N95 amplitude also showed a significant relationship with average RNFL and GCIPL thickness. In this study, we likewise confirmed a positive correlation between average RNFL thickness and FF PERG N95 amplitude. We found N95 amplitude and GCIPL thickness to be positively correlated as well. Lee et al.^31^ noted that N95 amplitude is correlated with the thickness of the GCIPL as well as the density of blood vessels in the peripapillary and macular regions of the retina. Other, more recent studies have demonstrated an association of macular vessel density with N95 amplitude, particularly in early-stage glaucoma^32,33^, as well as a strong association between parapapillary choroidal microvasculature dropout and decreased N95 amplitude ^33^. It is apparent that the conclusions of these earlier investigations are consistent with those of the current study. In terms of AUROC performance, the PERG N95 amplitude-related parameters performed better in this study than the SAP parameters. Comparison of the SD-OCT-derived structural parameters with the PERG parameters, however, indicated that the former still showed strong diagnostic abilities. This might be an intrinsic restriction of PERG, or it could be surmounted by controlling the variables influencing PERG variability. This concern might be addressed in the future by further studies employing progression analysis and lowering PERG variability.

PERG has a wide range of beneficial applications; however, it cannot be regarded as a totally dependable indicator, due to its changing amplitude, which is influenced by IOP ^34^ and inner-retinal thickness variation ^35^. Factors associated with IOP were not eliminated in this study, because individuals receiving medication for IOP control were included. Sub-analyses that did not include the 12 PPG patients who were on IOP medication were done in order to assess this. After excluding those 12 PPG patients, the IOP values in the PPG group showed no significant difference relative to the normal controls (Supplementary Table 1). The PPG group exhibited significantly lower FF PERG N95 amplitudes than did the normal controls, but no other noteworthy changes were observed in the PERG P50 or other HF amplitude values (Supplementary Table 2). The PPG group displayed noticeably thinner/thicker HF RNFL and GCIPL thickness ratios, however, in contrast to the original analysis, where there was only borderline significance for the smaller/larger HF N95 ratio with the post-hoc power of 0.501 (*P* = 0.069, Supplementary Table 3). The smaller/larger HF N95 amplitude ratio demonstrated a significant positive correlation with average GCIPL thickness in the Pearson’s correlation coefficient analysis (r = 0.345, *P* = 0.011, Supplementary Table 4), and showed a borderline significant positive correlation with affected HF average GCIPL thickness (r = 0.428, *P* = 0.053, post-hoc power = 0.952, Supplementary Table 4). The limited size of the patient group following the exclusion of those using IOP-lowering medications might explain these sub-analysis results. It would be intriguing in the future to attempt a comparison of different amplitude ratios such as the nasal/temporal HF ratio, which was examined using multifocal ERG in a prior study^36^, while adjusting for a number of other factors such as IOP and others.

This study has several limitations. First, PERG results are sometimes variable. Fixation shifts and stray light effects during stimulation might occur, even though the subject’s fixation is monitored by a trained observer. Testing time tends to vary from 10 to 25 min, depending on patient compliance, electrode stability, and the number of tests. Age could also affect results, with overall reductions in amplitude. PPG patients taking medicine to lower their IOP were included in this study, and those lower IOP values may also have impacted the outcomes. Secondly, as this study was of cross-sectional design, long-term prospective research, including examination of conversion from PPG to perimetric glaucoma, may add more information.

In conclusion, further to the present structural testing, PERG N95 amplitude and the HF ratio of N95 amplitude may offer useful information in terms of the functional evaluation of PPG. This study served to highlight the benefits of HF analysis in addition to the use of PERG N95 amplitude, based on which, it is proposed herein that combining those two could be a more effective diagnostic strategy.

## Methods

The Institutional Review Board of Seoul National University Hospital authorized the protocol for this prospective cross-sectional study (IRB no. 2208-130-1353), and all of the pertinent investigations and procedures followed the principles of the Declaration of Helsinki. Informed consent was obtained from all of the subjects.

### Study participants

In this study, the clinical data were gathered from glaucoma patients who had visited the Seoul National University Glaucoma Clinic between October 2022 and January 2023. The normal controls were volunteers who had come to the clinic in response to an advertisement. Patients who had glaucomatous optic discs (e.g., rim thinning, focal notching) or RNFL defects without any abnormal VF defect were allocated to the PPG group. Glaucomatous VF defect was defined as (1) glaucoma hemifield test values outside the normal limits or (2) three or more abnormal contiguous points with a probability of *P* < 0.05, of which at least one point has a probability of *P* < 0.01 on a pattern deviation plot, or (3) a pattern standard deviation (PSD) of *P* < 0.05. The additional inclusion requirements were as follows: (1) BCVA of 20/30 or better, (2) IOP ≤ 21 mmHg, (3) open anterior chamber angle on gonioscopy, and (4) AXL less than 26 mm. Patients with any history of retinal degeneration, retinal vein occlusion, or diabetic retinopathy were excluded. If both eyes met the criteria for inclusion, computer-based random selection of one eye per person was performed. The normal control group was defined as subjects having an IOP ≤ 21 mmHg, no prior ocular diseases or surgeries, no history of brain disorders, no signs of glaucomatous disc appearance, no abnormal RNFL changes on OCT, and no abnormal VF change on SAP.

Every participant had undergone complete ophthalmologic examinations, which included BCVA, IOP measurements by Goldmann applanation tonometry (Haag-Streit, Koniz, Switzerland), gonioscopy, refractive error measurements with an autorefractor (KR-890; Topcon Corporation, Tokyo, Japan), corneal pachymetry (Pocket II Pachymeter Echo Graph; Quantel Medical, Clermont-Ferrand, France), slit-lamp biomicroscopy, dilated fundus examination, SDP, red-free RNFL photography (Visucam 524; Carl Zeiss Meditec, Dublic, CA, USA), AXL measurements (Axis II PR; Quantel Medical, Inc., Bozeman, MT, USA), SAP with the Swedish interactive threshold algorithm according to the central 24-2 standard program (Humphrey Field Analyzer II; Carl Zeiss Meditec), Cirrus SD-OCT version 6.0 (Carl Zeiss Meditec) and PERG (Neuro- electroretinogram (ERG); Neurosoft, Ivanovo, Russia).

### Optical coherence tomography

Using Cirrus SD-OCT version 6.0, the RNFL thickness was determined in the optic Disc Cube 200 × 200 scan mode, and the GCIPL thicknesses were evaluated using GCA software (Carl Zeiss Meditec) and macular cube scanning. Only images with signal strengths greater than 6 were included. The average of the HF RNFL thickness was determined from the thickness values obtained from clock-hour sectors. Data from the 3 and 9 o’clock sectors were not taken into account, because they were not related to a single horizontal HF. The sectoral (superotemporal, superior, superonasal, inferonasal, inferior, and inferotemporal) thicknesses of the GCIPL were measured in an elliptical annulus around the fovea. The average of superior HF GCIPL thicknesses was determined in the macular cube scan mode from the superotemporal, superior, and superonasal thickness values. The inferior HF values were obtained in the same way.

Three glaucoma experts (YIS, EJA, and KHP) assessed the results of the ophthalmologic tests, including SD-OCT, for each participant. Affected HF was defined as one localized HF with localized RNFL defect or glaucomatous optic disc changes. Localized RNFL defect was identified as a dark wedge-shaped area on RNFL photography, with the tip linking the optic disc boundary in the exquisitely striated pattern of the surrounding RNFL ^37^. The aberrant glaucomatous optic disc changes included conspicuous neuroretinal rim thinning or notching on SDP. There were no cases of bilateral HF involvement. In accordance with the average thickness value acquired from SD-OCT, the HF with a thinner or thicker RNFL or GCIPL also was identified.

### Standard automated perimetry

Using the SAP central 24-2 test, MD and PSD were analyzed. Only VF exams with high reliability indices (fixation losses, false-positive and false-negative errors fewer than 20%) were included. The affected HF average total deviation was calculated using a total deviation plot in an area approximately corresponding to that assessed by HF PERG. More specifically, a rectangular area 30° × 15° above or below the field horizontal axis (17 points per HF) was employed to calculate the average of the deviations for either upper or lower HF^25^.

### Pattern electroretinography

The ERG stimulator (Neuro-ERG; Neurosoft, Ivanovo, Russia) used for PERG met the requirements of the International Society of Clinical Electrophysiology and Vision (ISCEV) standard: the 2012 update^38^. PERG was carried out by one single, qualified examiner. Patients with non-dilated pupils were seated in a dimly-lit room, and four electrodes were applied—two 35-mm Ag/AgCl skin electrodes to the lower eyelids, with two ground electrodes at both earlobes—for stimulation. Black and white reversing checkerboards with 0.8° checks were presented on a 24-inch liquid crystal display (LCD) monitor at a 35° × 30° visual angle and a distance of 60 cm. Because LCD monitor brightness might fluctuate, care was taken when selecting the viewing angle. The positioning of each subject was carefully observed, as issues may arise if the individual’s eye level is not adjusted to the center of the screen. The contrast between the black and white squares was 98%, and the mean luminance of the checkerboards was 100 cd/m^2^. The checkerboards used reversal rates of 4 reversals per second. The participants focused on a target at a red fixed point in the middle of the monitor after having their refractive error properly corrected. Signals were band-pass filtered (1–50 Hz) and sampled at 10,000 Hz. Computerized artifact rejection was employed, and traces exceeding 100 μV were automatically rejected as artifacts.

Transient PERG produces two main peaks, P50 and N95, which correspond to a positive peak at 50 ms (P50) and a slow, broad negative component at about 95 ms (N95). The N35 amplitude trough (a modest initial negative component with a peak time of about 35 ms) to the P50 peak is used to calculate the P50 amplitude. The amplitude of the N95 peak is measured, according to ISCEV standards, by calculating the distance between the P50 peak and the N95 trough. The PERG test was performed binocularly and continuously in the order of FF, UF and LF. During the HF examinations, the subject fixated on a red midpoint, and the unstimulated field was kept dark gray. Fixation was monitored by a trained observer. FF, UF, and LF values were automatically reported separately following HF exams.

### Statistical analyses

To compare the clinical traits of the normal control and PPG groups in terms of SD-OCT, SAP, and PERG parameter differences, we employed the independent t-test for continuous variables and the Chi-square test for categorical variables. Pairwise Pearson’s correlation coefficients were used to reveal the correlations between the SD-OCT or SAP parameters and the PERG parameters. To evaluate the relationships between age and PERG N95 amplitude, linear regression models were fitted, and the coefficients (β) with 95% confidence intervals (CIs) were generated. For continuous parameters, the AUROC was determined. All of the statistical analyses were performed with SPSS software (version 25.0; IBM Corporation, Armonk, NY, USA) and MedCalc (version 20.0; MedCalc Software Inc., Mariakerke, Belgium). A P value of less than 0.05 was regarded as statistically significant. For results considered borderline significant, post-hoc power analyses were carried out using G*Power 3.1.9.7 (www.psycho.uni-duesseldorf.de/abteilungen/aap/gpower3, Germany).

### Supplementary Information

Supplementary Legends.

Supplementary Figure 1.

Supplementary Figure 2.

Supplementary Table 1.

Supplementary Table 2.

Supplementary Table 3.

Supplementary Table 4.

## Data Availability

Upon reasonable request, the data from the study will be available by the corresponding author.

## Abbreviations

RGCs: Retinal ganglion cells
OCT: Optical coherence tomography
ONH: Optic nerve head
RNFL: Retinal nerve fiber layer
PERG: Pattern electroretinogram or pattern electroretinography
SAP: Standard automated perimetry
VF: Visual field
PPG: Preperimetric glaucoma
HF: Hemifield
BCVA: Best-corrected visual acuity
AXL: Axial length
IOP: Intraocular pressure
FF: Full field
UF: Upper hemifield
LF: Lower hemifield
AUROC: Area under the receiver operating characteristic curves
SD-OCT: Spectral-domain optical coherence tomography
SDP: Stereoscopic disc photography
GCIPL: Ganglion cell-inner plexiform layer
MD: Mean deviation
PSD: Pattern standard deviation
ERG: Electroretinogram
ISCEV: International Society of Clinical Electrophysiology and Vision
LCD: Liquid crystal display
CIs: Confidence intervals
