# Supplementary material for: Hemifield-based analysis of pattern electroretinography in normal subjects and patients with preperimetric glaucoma
Source: Sci Rep. 2024 Mar 1;14:5116. doi: 10.1038/s41598-024-55601-9 (PMC10907379; doi:10.1038/s41598-024-55601-9)
Supplement: Supplementary file 1 — Supplementary Legends. [file 41598_2024_55601_MOESM1_ESM.docx]

**Supplementary Figure 1. Correlation Between Pattern Electroretinogram Parameters and Spectral-domain Optical Coherence Tomography Parameters.**

**Supplementary Figure 2. Areas Under the Receiver Operating Characteristic Curve (AUROC) for Discriminating Preperimetric Glaucoma from Normal Control.**
