# Supplementary figures and images for: Hemifield-based analysis of pattern electroretinography in normal subjects and patients with preperimetric glaucoma
Source: Sci Rep. 2024 Mar 1;14:5116. doi: 10.1038/s41598-024-55601-9 (PMC10907379; doi:10.1038/s41598-024-55601-9)

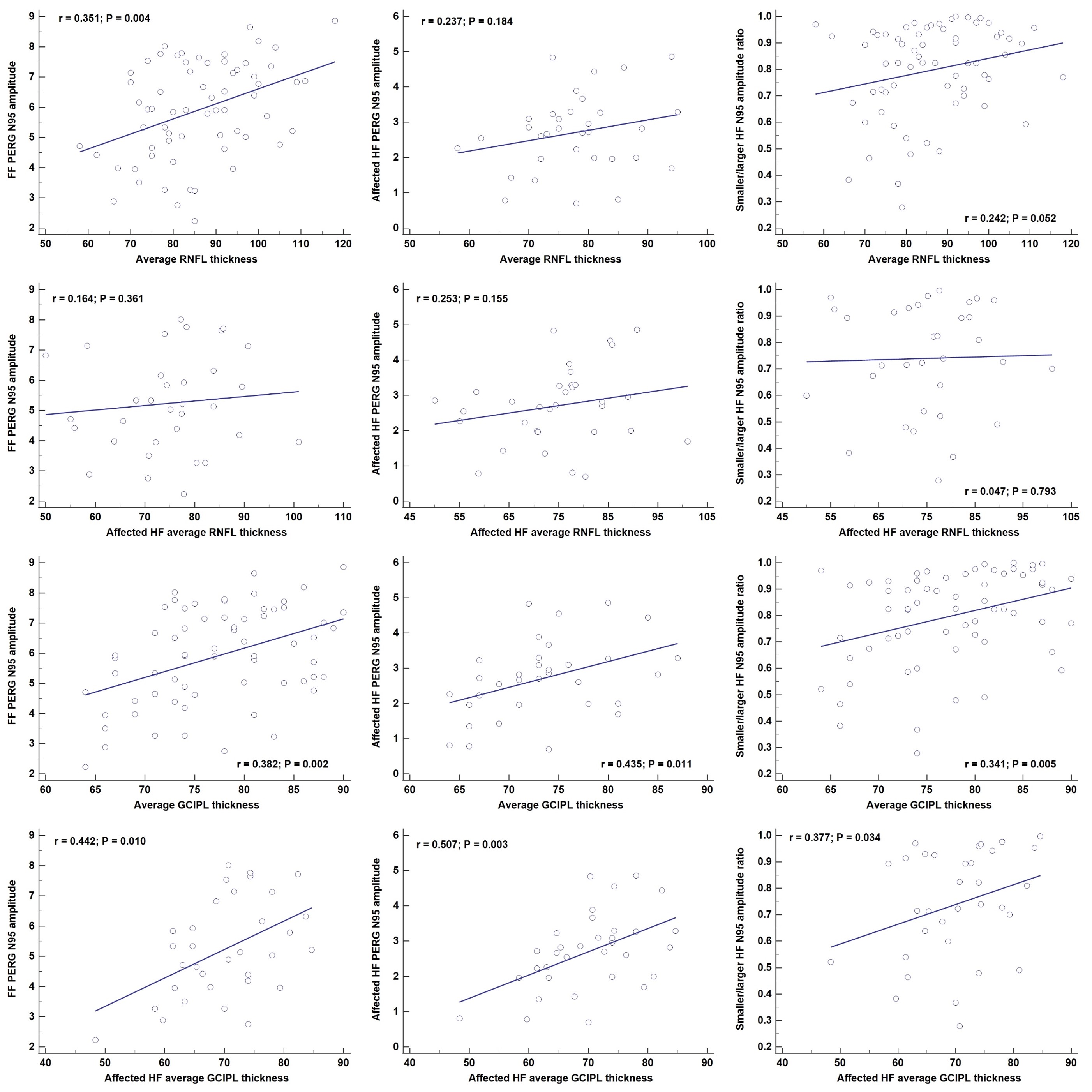

Supplement: Supplementary file 2 — Supplementary Figure 1. [file 41598_2024_55601_MOESM2_ESM.jpg]

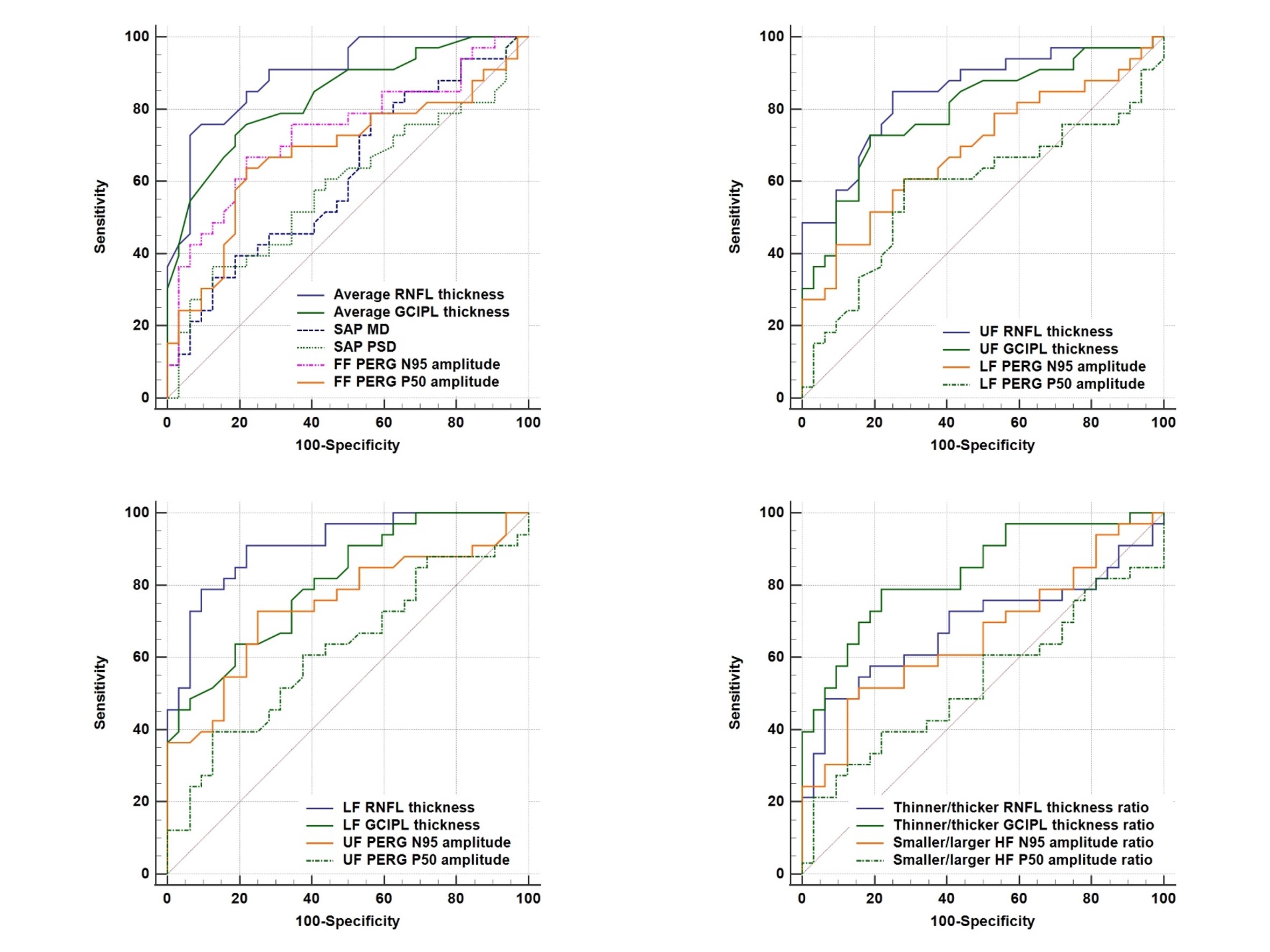

Supplement: Supplementary file 3 — Supplementary Figure 2. [file 41598_2024_55601_MOESM3_ESM.jpg]
