# Supplementary material for: Hemifield-based analysis of pattern electroretinography in normal subjects and patients with preperimetric glaucoma
Source: Sci Rep. 2024 Mar 1;14:5116. doi: 10.1038/s41598-024-55601-9 (PMC10907379; doi:10.1038/s41598-024-55601-9)
Supplement: Supplementary file 4 — Supplementary Table 1. [file 41598_2024_55601_MOESM4_ESM.docx]

**Supplementary Table 1. Participants’ Characteristics in Normal controls and Preperimetric Glaucoma (PPG) Patients without Glaucoma Medication**

| Characteristics | Control group (N=32) | PPG group without glaucoma medication (N=21) | *p*-Value |
| --- | --- | --- | --- |
| Age (years) | 48.63 ± 7.30 | 50.90 ± 11.54 | 0.500^a^ |
| Gender (n, %) |  |  |  |
| Male | 13 (40.6) | 12 (57.1) | 0.272^b^ |
| Female | 19 (59.4) | 9 (42.9) |  |
| BCVA (logMAR) | 0.00 ± 0.02 | −0.01 ± 0.08 | 0.827^a^ |
| Spherical equivalent | −2.54 ± 2.67 | −3.54 ± 2.86 | 0.270^a^ |
| Central corneal thickness (μm) | 547.50 ± 29.45 | 538.18 ± 30.09 | 0.389^a^ |
| Axial length (mm) | 24.36 ± 1.63 | 25.15 ± 1.39 | 0.118^a^ |
| Intraocular pressure (mmHg) | 15.53 ± 2.79 | 14.62 ± 2.99 | 0.279^a^ |
| SD-OCT |  |  |  |
| Average RNFL thickness (μm) | 94.69 ± 10.07 | 79.19 ± 8.44 | **<0.001**^a^ |
| Average GCIPL thickness (μm) | 81.53 ± 5.39 | 74.29 ± 6.37 | **<0.001**^a^ |
| SAP |  |  |  |
| MD (dB) | −1.73 ± 1.30 | −1.62 ± 1.64 | 0.665^a^ |
| PSD (dB) | 1.66 ± 0.37 | 1.76 ± 0.41 | 0.377^a^ |
| Affected HF average total deviation (dB) | N/A | −1.73 ± 1.62 | N/A |

PPG = preperimetric glaucoma; BCVA = best-corrected visual acuity; logMAR = logarithm of the minimum angle of resolution; SD-OCT = spectral-domain optical coherence tomography; RNFL = retinal nerve fiber layer; GCIPL = ganglion cell–inner plexiform layer; SAP = standard automated perimetry; MD = mean deviation; PSD = pattern standard deviation; HF = hemifield

Values are mean ± standard deviations.

Bold indicates that the P value reached statistical significance (<0.05).

^a^ Mann-Whitney test

^b^ Chi-square test
