# Supplementary material for: Hemifield-based analysis of pattern electroretinography in normal subjects and patients with preperimetric glaucoma
Source: Sci Rep. 2024 Mar 1;14:5116. doi: 10.1038/s41598-024-55601-9 (PMC10907379; doi:10.1038/s41598-024-55601-9)
Supplement: Supplementary file 5 — Supplementary Table 2. [file 41598_2024_55601_MOESM5_ESM.docx]

**Supplementary Table 2. Pattern Electroretinogram (PERG) Parameters in Normal controls and Preperimetric Glaucoma (PPG) Patients without Glaucoma Medication**

| Characteristics | Control group (N=32) | PPG group without glaucoma medication (N=21) | *p*-Value |
| --- | --- | --- | --- |
| PERG N95 Amplitude (μV) |  |  |  |
| Full field (FF) | 6.62 ± 1.26 | 5.69 ± 1.60 | **0.037** |
| Upper field (UF) | 3.30 ± 0.74 | 2.93 ± 0.92 | 0.098 |
| Lower field (LF) | 3.53 ± 0.76 | 3.19 ± 1.06 | 0.167 |
| PERG P50 Amplitude (μV) |  |  |  |
| Full field (FF) | 3.65 ± 0.98 | 3.08 ± 1.33 | 0.073 |
| Upper field (UF) | 1.87 ± 0.57 | 1.78 ± 0.81 | 0.548 |
| Lower field (LF) | 2.00 ± 0.57 | 2.03 ± 0.83 | 0.985 |
| PERG N95 Amplitude ratio |  |  |  |
| UF/FF | 0.51 ± 0.11 | 0.53 ± 0.13 | 0.151 |
| LF/FF | 0.54 ± 0.11 | 0.59 ± 0.18 | 0.317 |
| UF/LF | 0.95 ± 0.21 | 1.01 ± 0.46 | 0.964 |
| LF/UF | 1.10 ± 0.23 | 1.19 ± 0.64 | 0.913 |
| PERG P50 Amplitude ratio |  |  |  |
| UF/FF | 0.55 ± 0.23 | 0.65 ± 0.36 | 0.373 |
| LF/FF | 0.60 ± 0.29 | 0.71 ± 0.35 | 0.084 |
| UF/LF | 0.99 ± 0.37 | 0.95 ± 0.44 | 0.623 |
| LF/UF | 1.18 ± 0.64 | 1.33 ± 0.81 | 0.623 |

PERG = pattern electroretinogram; PPG = preperimetric glaucoma; FF = full field; UF = upper field; LF = lower field

Values are mean ± standard deviations.

Bold indicates that the P value reached statistical significance (<0.05).
