# Supplementary material for: Hemifield-based analysis of pattern electroretinography in normal subjects and patients with preperimetric glaucoma
Source: Sci Rep. 2024 Mar 1;14:5116. doi: 10.1038/s41598-024-55601-9 (PMC10907379; doi:10.1038/s41598-024-55601-9)
Supplement: Supplementary file 6 — Supplementary Table 3. [file 41598_2024_55601_MOESM6_ESM.docx]

**Supplementary Table 3. Comparison of Hemifield (HF) Ratios of Spectral-Domain Optical Coherence Tomography (SD-OCT) and Pattern Electroretinogram (PERG) Parameters in Normal controls and Preperimetric Glaucoma (PPG) Patients without Glaucoma Medication**

| Characteristics | Control group (N=32) | PPG group without glaucoma medication (N=21) | *p*-Value |
| --- | --- | --- | --- |
| SD-OCT |  |  |  |
| Affected/Unaffected HF RNFL thickness ratio | N/A | 0.85 ± 0.12 | N/A |
| Affected/Unaffected HF GCIPL thickness ratio | N/A | 0.93 ± 0.06 | N/A |
| Thinner/Thicker HF RNFL thickness ratio | 0.92 ± 0.06 | 0.85 ± 0.11 | **0.008** |
| Thinner/Thicker HF GCIPL thickness ratio | 0.98 ± 0.02 | 0.92 ± 0.09 | **<0.001** |
| PERG |  |  |  |
| Affected/Unaffected HF N95 amplitude ratio | N/A | 1.13 ± 0.64 | N/A |
| Affected/Unaffected HF P50 amplitude ratio | N/A | 0.99 ± 0.47 | N/A |
| Smaller/Larger HF N95 amplitude ratio | 0.86 ± 0.12 | 0.78 ± 0.20 | 0.069 |
| Smaller/Larger HF P50 amplitude ratio | 0.76 ± 0.16 | 0.76 ± 0.26 | 0.996 |

HF = hemifield; SD-OCT = spectral-domain optical coherence tomography; PERG = pattern electroretinogram; PPG = preperimetric glaucoma; RNFL = retinal nerve fiber layer; GCIPL = ganglion cell–inner plexiform layer

Affected HF was defined as a localized one-HF with localized RNFL defect or glaucomatous optic disc changes.

Values are mean ± standard deviations.

Bold indicates that the P value reached statistical significance (<0.05).
