# Supplementary material for: Hemifield-based analysis of pattern electroretinography in normal subjects and patients with preperimetric glaucoma
Source: Sci Rep. 2024 Mar 1;14:5116. doi: 10.1038/s41598-024-55601-9 (PMC10907379; doi:10.1038/s41598-024-55601-9)
Supplement: Supplementary file 7 — Supplementary Table 4. [file 41598_2024_55601_MOESM7_ESM.docx]

**Supplementary Table 4. Correlation Between Pattern Electroretinogram (PERG) Parameters and Spectral-Domain Optical Coherence Tomography (SD-OCT)/ Standard Automated Perimetry (SAP) Parameters in Normal controls and Preperimetric Glaucoma (PPG) Patients without Glaucoma Medication**

|  | FF PERG N95 amplitude | | Affected HF PERG N95 amplitude | | Smaller/larger HF N95 amplitude ratio | |
| --- | --- | --- | --- | --- | --- | --- |
|  | r | *p*-Value | r | *p*-Value | r | *p*-Value |
| Average RNFL thickness | 0.377 | **0.005** | 0.336 | 0.137 | 0.234 | 0.092 |
| Affected HF average RNFL thickness | 0.319 | 0.159 | 0.360 | 0.108 | 0.163 | 0.479 |
| Average GCIPL thickness | 0.311 | **0.024** | 0.390 | 0.080 | 0.345 | **0.011** |
| Affected HF average GCIPL thickness | 0.366 | 0.103 | 0.452 | **0.040** | 0.428 | 0.053 |
| SAP MD | −0.041 | 0.768 | −0.183 | 0.426 | 0.132 | 0.347 |
| Affected HF average total deviation | −0.082 | 0.732 | −0.180 | 0.466 | 0.349 | 0.132 |

PERG = pattern electroretinogram; SD-OCT = spectral-domain optical coherence tomography; SAP = standard automated perimetry; PPG = preperimetric glaucoma; FF = full field; HF = hemifield; RNFL = retinal nerve fiber layer; GCIPL = ganglion cell–inner plexiform layer; MD = mean deviation

Values are mean ± standard deviations.

Bold indicates that the P value reached statistical significance (<0.05).
